# Psychometric analysis of the new ADHD DSM-V derived symptoms

**DOI:** 10.1186/1471-244X-12-21

**Published:** 2012-03-20

**Authors:** Ahmad Ghanizadeh

**Affiliations:** 1 Research Center for Psychiatry and Behavioral Sciences, School of Medicine, Shiraz University of Medical Sciences, Shiraz, Iran; 2 Department of Psychiatry, School of Medicine, Shiraz University of Medical Sciences, Shiraz, Iran

**Keywords:** ADHD, DSM-IV, DSM-V, Revision, Subtype, Factor analysis, Reliability, Validity, Farsi

## Abstract

**Background:**

Following the agreements on the reformulating and revising of ADHD diagnostic criteria, recently, the proposed revision for ADHD added 4 new symptoms to the hyperactivity and Impulsivity aspect in DSM-V. This study investigates the psychometric properties of the proposed ADHD diagnostic criteria.

**Method:**

ADHD diagnosis was made according to DSM-IV. The parents completed the screening test of ADHD checklist of Child Symptom Inventory-4 and the 4 items describing the new proposed symptoms in DSM-V.

**Results:**

The confirmatory factor analysis of the ADHD DSM-V derived items supports the loading of two factors including inattentiveness and hyperactivity/impulsivity. There is a sufficient reliability for the items. However, confirmatory factor analysis showed that the three-factor model is better fitted than the two-factor one. Moreover, the results of the exploratory analysis raised some concerns about the factor loading of the four new items.

**Conclusions:**

The current results support the two-factor model of the DSM-V ADHD diagnostic criteria including inattentiveness and hyperactivity/impulsivity. However, the four new items can be considered as a third factor.

## Background

Attention-deficit/hyperactivity disorder (ADHD) is one of the most common behavioral disorders in children and adolescents. Its rate in community samples is variably reported. A study reported the rate of 5.29% [1]. Meanwhile, the rate of its screening symptoms is much higher, reaching up to 10.1% in school age children [2]. This high rate of ADHD prevalence emphasizes the need for accurate identification and diagnosis of ADHD [3].

There has been a recent significant argument or controversy regarding the necessity of reformulating and revising ADHD criteria [1,4,5]. For example, recent criticism of the current ADHD subtypes and the suggestion of including age-specific ADHD criteria in DSM V should be considered [6]. In addition, the current ADHD subtypes are frequently criticized [3]. Some researchers are interested in introducing ADHD-inattentive type as a learning disorder [7]. Furthermore, there is a debate whether oppositional defiant disorder should be considered as a type of ADHD [8,9]. Girls with ADHD are underdiagnosed in the community [6]. Moreover, the impact of the change in the age of the onset has been investigated [10].

Given that the proposed DSM-V criteria for ADHD are available and would be implemented in the near future [11], it is advised that their psychometric properties and modifications be studied before their clinical application. To the best of the author's knowledge, there are no published studies investigating the psychometric properties of the proposed ADHD diagnostic criteria for DSM-V.

DSM-IV defines ADHD as a cluster of symptoms; the patient must have at least six or more out of the 9 symptoms of inattention and/or six or more out of the 9 symptoms of hyperactivity/impulsivity [12]. The proposed revision of ADHD by American Psychiatric Association added 4 new symptoms to the Hyperactivity and Impulsivity aspect in DSM- V. These four symptoms are: "Tends to act without thinking, such as starting tasks without adequate preparation or avoiding reading or listening to instructions, may speak out without considering consequences or make important decisions on the spur of the moment, such as impulsively buying items, suddenly quitting a job, or breaking up with a friend", "Is often impatient, as shown by feeling restless when waiting for others and wanting to move faster than others, wanting people to get to the point, speeding while driving, and cutting into traffic to go faster than others", "Is uncomfortable doing things slowly and systematically and often rushes through activities or tasks", and "Finds it difficult to resist temptations or opportunities, even if it means taking risks (A child may grab toys off a store shelf or play with dangerous objects; adults may commit to a relationship after only a brief acquaintance or take a job or enter into a business arrangement without doing due diligence)" [11].

The aim of this study was to investigate the psychometric properties of the proposed ADHD symptoms in DSM-V. In the first step, factor analyses were conducted to assess the loadings for the symptoms. Then, the convergent and discriminative validity of the categories of inattentiveness and hyperactivity-impulsivity of DSM-V ADHD symptoms were assessed. Finally, the internal reliability of the inattentiveness and hyperactivity- impulsivity was calculated.

## Methods

106 children, who were consecutive referrals to a university affiliated Child and Adolescent Psychiatry Clinic in Shiraz, Iran, participated in this study. All of the children and adolescents were interviewed face to face by a board certified Child and Adolescent psychiatrist. In addition, at least one of their parents or caregivers was interviewed face to face as a collateral information resource.

The Diagnostic and Statistical Manual of Mental Disorders, Fourth Edition, DSM-IV diagnostic criteria was used to make psychiatric diagnoses [12]. Interviews were conducted according to the Farsi version of the Schedule for Affective Disorders and Schizophrenia for School-Age Children [13].

Parents reported ADHD symptoms by completing the ADHD checklist of child symptom inventory-4 [14-16]. The ADHD checklist of child symptom inventory-4 includes 18 symptoms. The symptoms are categorized into two groups of inattentiveness and hyperactive/impulsivity symptoms. The inattentiveness symptoms category consists of 9 symptoms according to DSM-IV. The category of hyperactive/impulsivity symptoms consists of 9 symptoms according to DSM-IV as well. In fact, the symptoms are the DSM-IV diagnostic criteria. There is a 5-point Likert response scale for the symptoms. The responses ranged from "never," "sometimes," "often," to "almost always". Scores 0 and 1 were assigned to the categories of "never" and "sometimes", respectively. The categories of "often" and "almost always" were assigned to 2 and 3, respectively. The range of scoring for each of inattentiveness and hyperactivity-impulsivity categories was from 0 to 9. The Farsi version of this checklist has enough reliability, convergent and discrimination validity [15] and has been used in many studies [17-19]. The internal reliability of this checklist for ADHD-inattentive type, ADHD-Hyperactive impulsive type, and combined type of ADHD is 0.81, 0.85, and 0.83, respectively [14].

The four new items proposed by DSM-V to be added to ADHD diagnostic criteria were translated into Farsi and back translated into English by a bilingual child and adolescent psychiatrist and a psychologist. Every effort was made to preserve the concept of each symptom. After a pilot study on children referred to the clinic, the final version was used in the current study. The responses to these symptoms were in the Likert scale ranging from "never," "sometimes," "often," to "almost always".

The children and parents or caregivers gave their assent or informed written consent for voluntary participation in this study. This study was approved by the Ethics Committee of Shiraz University of Medical Sciences.

### Analysis

SPSS statistical software was used to analyze the data. A factor analysis with varimax rotation was conducted to examine the factor structure of the ADHD DSM-V symptoms. The Kaiser-Meyer-Olkin Measure and the Bartlett's test of sphericity were conducted. Internal consistency was examined using Cronbach's tests.

One-, two-, three-factor models of confirmatory factor analysis were also conducted using LISREL 8.54 software. The convergent and discriminative validity of ADHD symptoms were analyzed using Pearson's r correlation coefficient.

Another factor analysis was also conducted including the four newly proposed symptoms to examine item loading of the 13 symptoms of DSM-V derived hyperactivity- impulsivity symptoms. Here, the symptoms of inattentiveness were not included in the analysis. This analysis was conducted to examine whether the 13 items could be divided into two categories of hyperactivity and impulsivity.

Another factor analysis was conducted including the DSM-IV derived inattentiveness symptoms and the four new symptoms proposed in DSM-V. The symptoms of hyperactivity-impulsivity of DSM-IV were not included.

## Results

The sample included 84 (79.2%) boys and 22 (20.8%) girls. The age range of the children and adolescents was 5.5 to 17years. Their mean age was 9.1(SD = 2.5) years.

The Kaiser-Meyer-Olkin Measure was 0.76. It shows the adequacy of sampling. The Bartlett's test of sphericity was less than 0.001. These results indicate that the data are suitable for factor analysis. The factor loading of the principal component analysis is indicated in Table 1. The factor of Hyperactivity-Impulsivity explained 30.4% (eigenvalue = 6.7) of the total variance. The factor of Inattentiveness accounted for 12.1% (eigenvalue = 2.6). Nearly all of the symptoms of inattentiveness were loaded in one factor. All of the Hyperactivity-Impulsivity symptoms were loaded on another factor. Three out of the four newly proposed ADHD separate symptoms were loaded on the factor including inattentiveness symptoms.

**Table 1:** Principal Component Analysis of the ADHD DSM-V Checklist by Rotated Method of Varimax

Component	DSM-V symptoms Hyperactivity- Impulsivity	
**Inattentiveness**		
ADHD- item 1- makes careless mistakes	-.049	.600
ADHD- item 2- sustaining attention ADHD- item 3- listening when spoken to	.032 .323	.731 .319
ADHD- item 4- follows instructions	.354	.515
ADHD- Item 5- organizing tasks	.164	.775
ADHD-Item 6 - sustained mental effort	-.097	.784
ADHD- item 7- loses things	.185	.527
ADHD- item 8- distracted by extraneous stimuli	.223	.536
ADHD- item 9- forgetful in daily activities	.157	.486
ADHD- item10- fidgets with hands	.532	.227
ADHD- item11- leaves seat in classroom	.657	.206
ADHD- item 12- runs about	.638	.178
ADHD- item 13- playing or leisure activities	.864	-.013
ADHD- item 14- often "on the go"	.800	-.008
ADHD- item 15- talks excessively	.726	.152
ADHD- item 16- blurts out answers	.663	.134
ADHD- item 17- awaiting turn	.625	.335
ADHD- item 18- interrupts or intrudes on others	.713	.086
ADHD- item 19- act without thinking	.272	.412
ADHD- item 20- impatient	.431	.330
ADHD- item 21- uncomfortable doing things slowly and systematically	.358	.430
ADHD- item 22- difficult to resist temptations or opportunities	.236	.399

Extraction Method: Principal Component Analysis. Rotation Method: Varimax with Kaiser Normalization.

In order to test which of the various models gives the best fit to the data, three confirmatory factor analyses were conducted. A one-factor model was not a good fit (Chi- square = 384.65, df = 209, P value 0.0001, Root Mean Square Error of Approximation (RMSEA) = 0.098, Non-normed Fit index (NNFI) = 0.96, Comparative Fit index = 0.96.).

A two-factor model fit well. The results of two-factor model confirmatory factor analysis showing the correlation between inattentiveness and hyperactivity/impulsivity factors that was .56 are displayed in Table 2.

**Table 2:** The two-factor model of Confirmatory Factor Analysis of the ADHD DSM- V Checklist

Component	DSM-V symptoms Hyperactivity- Impulsivity
**Inattentiveness**	
ADHD- item 1- makes careless mistakes	.49
ADHD- item 2- sustaining attention	.71
ADHD- item 3- listening when spoken to ADHD- item 4- follows instructions	.53 .73
ADHD- Item 5- organizing tasks	.81
ADHD-Item 6 - sustained mental effort	.66
ADHD- item 7- loses things	.57
ADHD- item 8- distracted by extraneous stimuli	.63
ADHD- item 9- forgetful in daily activities	.56
ADHD- item10- fidgets with hands	.64
ADHD- item11- leaves seat in classroom	.72
ADHD- item 12- runs about	.71
ADHD- item 13- playing or leisure activities	.84
ADHD- item 14- often "on the go"	.81
ADHD- item 15- talks excessively	.76
ADHD- item 16- blurts out answers	.69
ADHD- item 17- awaiting turn	.76
ADHD- item 18- interrupts or intrudes on others	.72
ADHD- item 19- act without thinking	.49
ADHD- item 20- impatient	.62
ADHD- item 21- uncomfortable doing things slowly and systematically	.58
ADHD- item 22- difficult to resist temptations or opportunities	.51

Chi-square = 384.65, df = 209, P valu < 0.0001, Root Mean Square Error of Approximation (RMSEA) = 0.098, Non-normed Fit index (NNFI) = 0.96, Comparative Fit index = 0.96.

However, a three-factor model of confirmatory factor analysis also fit well and it was better than the two-factor model (Table 3).

**Table 3:** The three-factor model of Confirmatory Factor Analysis of the ADHD DSM-V Checklist

Component Newly	DSM-V symptoms Hyperactivity- Impulsivity
**Inattentiveness added items**	
ADHD- item 1- makes careless mistakes	.49
ADHD- item 2- sustaining attention	.71
ADHD- item 3- listening when spoken to	.52
ADHD- item 4- follows instructions	.72
ADHD- Item 5- organizing tasks	.82
ADHD-Item 6 - sustained mental effort	.67
ADHD- item 7- loses things	.57
ADHD- item 8- distracted by extraneous stimuli	.63
ADHD- item 9- forgetful in daily activities	.56
ADHD- item10- fidgets with hands	.65
ADHD- item11- leaves seat in classroom	.74
ADHD- item 12- runs about	.73
ADHD- item 13- playing or leisure activities	.86
ADHD- item 14- often "on the go"	.83
ADHD- item 15- talks excessively	.78
ADHD- item 16- blurts out answers	.71
ADHD- item 17- awaiting turn	.78
ADHD- item 18- interrupts or intrudes on others	.74
ADHD- item 19- act without thinking	.63
ADHD- item 20- impatient	.80
ADHD- item 21- uncomfortable doing things slowly and systematically	.78
ADHD- item 22- difficult to resist temptations or opportunities	.66

Chi-square = 31.84, df = 206, P valu < 0.0001, Root Mean Square Error of Approximation (RMSEA) = 0.077, Non-normed Fit index (NNFI) = 0.99, Comparative Fit index = 0.99.

The factor loading of the second component analysis including only the symptoms of hyperactivity-impulsivity of DSM-V is displayed in Table 4. The Kaiser-Meyer-Olkin Measure was 0.83. Bartlett's test of sphericity was less than 0.001. It shows that all of the symptoms of the ADHD DSM-IV derived are loaded in one factor. Meanwhile, the four new symptoms proposed in DSM-V are loaded in another factor.

**Table 4:** Principal components analysis of the hyperactivity-impulsivity symptoms of ADHD DSM-V Checklist

Hyperactivity-impulsivity symptoms		
	Component	
	1	2
ADHD- item10- fidgets with hands	.566	.123
ADHD- item11- leaves seat in classroom	.666	.214
ADHD- item 12- runs about	.629	.225
ADHD- item 13- playing or leisure activities	.834	.111
ADHD- item 14- often "on the go"	.771	.157
ADHD- item 15- talks excessively	.753	.154
ADHD- item 16- blurts out answers	.682	.112
ADHD- item 17- awaiting turn	.574	.396
ADHD- item 18- interrupts or intrudes on others	.717	.132
ADHD- item 19- act without thinking	.207	.496
ADHD- item 20- impatient	.208	.794
ADHD- item 21- uncomfortable doing things slowly and systematically	.187	.755
ADHD- item 22- difficult to resist temptations or opportunities	.022	.781

Extraction Method: Principal Component Analysis. Rotation Method: Varimax with Kaiser Normalization.

The principal component analysis including the DSM-IV derived inattentiveness symptoms and the four new symptoms proposed in DSM-V indicated the two factor loading (Table 5). This analysis indicates that all of the inattentiveness symptoms are loaded in one factor and the new symptoms proposed in DSM-V are loaded in another factor.

**Table 5:** Principal component analysis including the DSM-IV derived inattentiveness symptoms and the four new symptoms proposed in DSM-V

Inattentiveness symptom of DSM-IV and new proposed symptoms in DSM-V		
	Component	
	1	2
ADHD- item 1- makes careless mistakes	.676	-.045
ADHD- item 2- sustaining attention	.780	.079
ADHD- item 3- listening when spoken to	.486	.122
ADHD- item 4- follows instructions	.551	.322
ADHD- Item 5- organizing tasks	.686	.386
ADHD-Item 6 - sustained mental effort	.667	.192
ADHD- item 7- loses things	.414	.341
ADHD- item 8- distracted by extraneous stimuli	.450	.367
ADHD- item 9- forgetful in daily activities	.579	.057
ADHD- item 19- act without thinking	.275	.565
ADHD- item 20- impatient	.069	.785
ADHD- item 21- uncomfortable doing things slowly and systematically	.136	.793
ADHD- item 22- difficult to resist temptations or opportunities	.058	.750

Extraction Method: Principal Component Analysis. Rotation Method: Varimax with Kaiser Normalization.

The convergent and discriminative validity for the whole 22 symptoms proposed for ADHD in DSM-V were calculated. The range of convergent validity for the symptoms of inattentiveness was from 0.504 to 0.772 and that of discriminative validity for the symptoms of inattentiveness was from 0.017 to 0.427. Also, the range of convergent validity for the symptoms of hyperactivity-impulsivity was from 0.42 to 0.770 and that of discriminative validity for the symptoms of hyperactivity-impulsivity was from 0.12 to 0.39.

The alpha coefficient for the whole 24 symptoms of ADHD in DSM-V was 0.88. The alpha for the DSM-V hyperactivity-impulsivity was 0.87. It was 0.80 for DSM-IV inattention.

## Discussion

To the best of the author's knowledge, this is the first study investigating psychometric and factor structure of ADHD DSM-V derived symptoms. So, it is not possible to compare the current results with those of other studies. Confirmatory factor analysis confirmed the proposed two-factor loading of inattentiveness and hyperactivity/impulsivity for the new ADHD DSM-V criteria. However, the three-factor model of confirmatory factor analysis showed that the four new items can be considered as the third factor.

The results indicate that convergent and discriminative validity for ADHD DSM-V derived inattention symptoms are sufficient. Although the symptoms of hyperactivity- impulsivity are discriminated from inattentiveness symptoms, the convergent validity of the four newly proposed symptoms in DSM-V is not as high as that of the 9 symptoms derived from DSM-IV. The three new criteria for hyperactivity/impulsivity were loaded in inattentiveness factor rather than in hyperactivity-impulsivity factor. These may not support the fact that the 4 proposed symptoms for revision of ADHD exactly describe hyperactivity-impulsivity symptoms. However, the internal consistency and reliability of the inattentiveness and hyperactive/impulsivity symptoms are high.

Considering the factor loading of the four newly proposed symptoms added to DSM-V, there is a concern that inattentiveness symptoms may falsely increase the diagnosis of ADHD-hyperactive/impulsive type or combined type of ADHD. It means that the symptoms which are loaded as inattentive symptoms may lead to subthreshold ADHD- hyperactive/impulsive type using DSM-IV, while fulfilling criteria of ADHD- hyperactive/impulsive type using DSM-V.

With respect to the fact that the better diagnoses and classification of children with ADHD could lead to a better treatment, more discussion and justification about the new items are required. Probably, future studies should investigate the neuropsychological functioning of children with ADHD for the classification of the subtypes of ADHD. The current results indicated that continued research is required to reach accurate diagnostic criteria for making accurate ADHD diagnoses.

There is some overlap between ADHD symptoms and ODD in DSM-IV [20]. ODD symptoms are properly differentiated from ADHD. However, two items of the ADHD including "Often has trouble organizing activities" and "Often runs about or climbs when and where it is not appropriate" are loaded in the oppositional defiant disorder component rather than ADHD component [20]. Another concern is whether the new added symptoms in DSM-V are well differentiated from ODD symptoms. This needs further studies.

There are some limitations in this study which need to be considered. This study was conducted on a clinical sample of children and adolescents with ADHD. Further studies with larger sample size including community sample with a wider age rage are recommended. The children and their parents were the sources of information. Including other informants such as teachers is also recommended. This study is based on one sample in a specific geographical area. In addition, the use of translation instead of the actual questionnaire is another limitation. A multi-site approach with a more limited age range would be required to appropriately assess the psychometric properties of the proposed items of a classification used worldwide.

Despite the above-mentioned limitations, this is the first study that assesses psychometric properties of ADHD DSM-V derived symptoms. In addition, the children, adolescents and parents were interviewed face to face using a well known semi- structured interview. Moreover, all the interviews were conducted by a Board-certified child and adolescent psychiatrist.

## Conclusion

The findings of present study support the two-factor model of the DSM-V ADHD diagnostic criteria including inattentiveness and hyperactivity/impulsivity. Nevertheless, the four new items can be considered as a third factor.

## Competing interests

The authors declare that they have no competing interests.

## Authors' contributions

All authors read and approved the final manuscript.

## Pre-publication history

The pre-publication history for this paper can be accessed here:

http://www.biomedcentral.com/1471-244X/12/21/prepub
